# Efficacy and safety of adalimumab in pediatric patients with Crohn’s disease: A systematic review and meta-analysis

**DOI:** 10.1007/s00228-023-03613-1

**Published:** 2023-12-29

**Authors:** Bin Chen, Zhuan Zou, Xiaoyan Zhang, Dongqiong Xiao, Xihong Li

**Affiliations:** 1 Department of Pediatrics, West China Second University Hospital, Sichuan University, Chengdu, 610000 China; 2 Key Laboratory of Birth Defects and Related Diseases of Women and Children, Sichuan University, Chengdu, 610000 China

**Keywords:** Adalimumab, Crohn’s disease, Child, Meta-analysis, Efficacy and safety

## Abstract

**Purpose:**

There is currently no curative treatment for childhood Crohn’s disease (CD). This meta-analysis aimed to validate the efficacy and safety of adalimumab (ADA) in pediatric patients with CD.

**Materials and methods:**

We searched all relevant studies in the PubMed, Web of Science, Embase, and Cochrane Library databases. The primary outcomes were induction (≤ 12 weeks) and maintenance (up to 48 weeks) of remission and response. Secondary outcomes were severe adverse events and opportunistic infections to ADA. The Cochrane bias assessment tool was used to assess the risk of bias in randomized controlled trials. The methodological quality of the single-arm studies was assessed using the methodological index for non-randomized studies tool.

**Results:**

Ten clinical trials involving a total of 885 patients were included. Results indicated that 59% (95% confidence interval [CI] 39–80%) of the subjects treated with ADA achieved induction of remission, and 60% (95% CI 35–86%) of the subjects treated with ADA achieved induction of response, 57% (95% CI 44–70%) achieved maintenance of remission, and 63% (95% CI 26–69%) achieved maintenance of response.

**Conclusion:**

Current evidence indicates that ADA is effective in children and adolescents with CD and that adverse events vary but are usually not severe.

**Systematic review registration:**

https://www.crd.york.ac.uk/prospero/, identifier CRD42023402199.

**Supplementary Information:**

The online version contains supplementary material available at 10.1007/s00228-023-03613-1.

## Introduction

Crohn’s disease (CD) is a chronic, relapsing disease of the gastrointestinal tract that results in significant morbidity and affects the quality of life. Nearly a quarter of people with CD develop it before the age of 20 [1, 2], and the most common age is school age and early adolescence [3]. Pediatric patients account for less than 1.5% of all prevalent inflammatory bowel disease cases [4]. Pediatric-onset CD is more extensive and severe at presentation, with a more aggressive disease course and worse prognosis when compared to adult-onset [5]. In addition to the clinical manifestations of recurrent abdominal pain, diarrhea, and anal lesions common to adult CD, pediatric-onset CD also has the characteristics of growth retardation, delayed puberty, and decreased body mass index. Additionally, it affects mental health and is associated with malnutrition and the need for surgery [6].

Treating CD is a significant challenge for clinicians because there is currently no curative treatment. Traditional therapy includes corticosteroids, immunomodulators, and exclusive enteral nutrition. However, when a patient does not respond to these first-line treatments, biologic therapies, such as anti-tumor necrosis factor (TNF)-α antibodies, are considered. Whether conventional or biological therapy, the end goal is to achieve endoscopic remission.

TNF molecules are homotrimers that exist either as membrane TNF (mTNF) on cell surfaces or as free molecules in solution (soluble TNF) following the cleavage of mTNF by TNF-α-converting enzyme (TACE). The possibility of the binding of mTNF or soluble TNF to TNF-R being inhibited has also been reported [2, 7]. TNF-α antibodies are a valid therapeutic option for pediatric patients with CD. The first TNF-α inhibitor that was approved for the treatment of CD in adults and children was infliximab (IFX), which is a monoclonal IgG1 antibody with a partially murine anti-TNF Fab region [8]. However, recent data indicate that approximately 30% of patients lose response within 3 years after starting the treatment, while half withdraw from the treatment due to a lack of the drug [9, 10]. In randomized clinical trials, adalimumab (ADA), an IgG1 antibody containing a humanized Fab region [11], has shown effectiveness in the treatment of pediatric patients with CD [12, 13], and has been approved by the United States Food and Drug Administration for treating moderate and severe CD in children. It is also recommended by the European consensus guidelines for the treatment of long-term active intestinal diseases in children [14]. Furthermore, several systematic reviews and meta-analyses have demonstrated that ADA is also safe and effective in the treatment of adult patients with CD [15–17]. A systematic review on this topic has already been reported. Particularly, a systematic review and meta-analysis conducted by Li et al. [18] revealed no significant differences between IFX and standard of care with respect to the maintenance of clinical remission at 6 months and 1 year. However, such a study on ADA has not yet been reported. Therefore, in this systematic review and meta-analysis, we aimed to examine the efficacy and safety of ADA in inducing and maintaining remission in pediatric patients with CD.

## Methods

The literature review was conducted according to the EQUATOR Network website, including the PRISMA 2020 statement [19].

### Search strategy and inclusion criteria

PubMed, Web of Science, Embase, and Cochrane Library were searched for clinical trials examining the effectiveness and safety of ADA in children with CD on January 6, 2023. The most recent search was conducted on November 3, 2023. Cited references of the retrieved articles and previous reviews were manually checked to identify additional eligible trials. The retrieved studies were imported into the EndNoteX9 software (Clarivate Analytics, London, UK). Thereafter, two researchers (C.B. and Z.C.) independently searched and screened the candidate articles, checking eligibility for inclusion. The keywords used as search terms were as follows: “Crohn disease,” “Crohn’s Disease,” “CD,” “Crohns disease,” “adalimumab,” “child,” “pediatric,” and “adolescent.” An example search strategy is presented in Fig. S1.

We developed a patient, intervention, comparison, outcome, and study design (PICOS) approach as the eligibility criteria. (1) Population: children and adolescents (2–18 years of age) with CD; children with ulcerative colitis or unclassified IBD were excluded. (2) Intervention: ADA was administered subcutaneously. (3) Comparison: no placebo-controlled trials have been conducted on CD treatment in children as this is considered unethical. (4) Outcome: the eligible clinical trials had to present data on induction of remission (defined as clinical remission pediatric CD activity index [PCDAI] score ≤ 10 after ≤ 12 weeks of treatment), induction of clinical response (defined as a PCDAI score < 30, and a decrease in PCDAI score ≥ 12.5 points from the baseline score, after at least 12 weeks of treatment), maintenance of remission (defined as clinical remission after at least 48 weeks of treatment), maintenance of response (defined as clinical response after ≤ 1 year of therapy), as the primary outcome. The secondary outcome was the incidence of adverse effects, such as infections, injection-related reactions, and serious adverse events; this was individually examined. (5) Study design: randomized controlled trials (RCTs) and retrospective or prospective cohort studies assessing predefined outcomes; case–control studies and conference abstract data were excluded. Discrepancies regarding study inclusion were resolved through discussions with the corresponding author (L.X.H.). Only published data were included in these studies. For duplicate publications of the same clinical trial, we selected the article with latest data.

### Date extraction and quality assessment

Two authors (Z.X.Y. and X.D.Q.) independently extracted relevant data from each included trials using a unified data form. The quality of the RCTs was assessed using the Cochrane Collaboration risk of bias assessment tool. Furthermore, the researchers evaluated the RCT studies item by item, and the evaluation results were expressed as low risk, high risk, or unclear [20]. The methodological quality of single-arm studies was assessed using the methodological index for non-randomized studies (MINORS) tool, which consists of eight items for noncomparative studies. An item was scored “0” when not reported, “1” when inadequately reported, and “2” when adequately reported. The maximum score was 16 [21].

### Statistical analysis

The proportions of outcomes were calculated for each treatment arm. Pooled weighted proportions were calculated after treatment with ADA using STATA software (Stata-Corp LLC, College Station, TX, USA). Heterogeneity across studies was tested by *I*^2^ statistic, and studies with an *I*^2^ value of > 50% were considered to have significant heterogeneity. Pooled estimates of the effect size and relevant 95% confidence interval (CI) for each treatment arm were obtained using the random or fixed effects model. Sensitivity analyses were conducted by excluding any single arm and its combinations to investigate their influence on pooled proportions. Publication bias was evaluated using the Egger test. A value of *P* < 0.05 was considered statistically significant. Furthermore, subgroup analyses were conducted to analyze the heterogeneity between studies.

## Results

### Summary of study characteristics

**Fig. 1:**
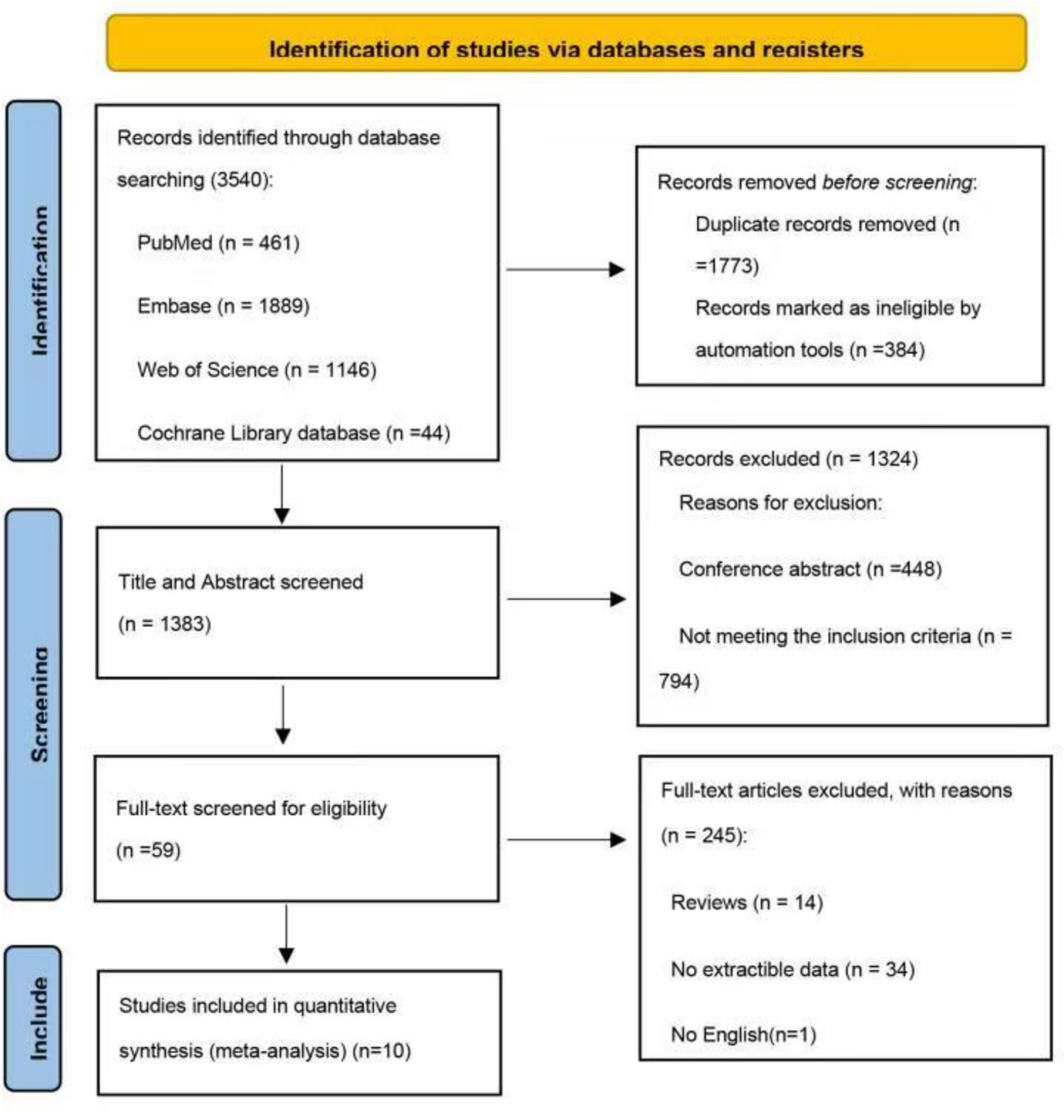
Flow chart of the literature search

A flow diagram showing the study selection process is provided in Fig. 1. Ten articles were considered eligible for inclusion in the systematic review. These included eight single-arm cohort studies and two RCTs [2, 13, 22–29]. The basic characteristics of the included studies are summarized in Table 1. Furthermore, most of these studies were conducted in Europe [2, 22, 23, 25, 27], North America [13, 28, 29], or both [24]; only one was conducted in Asia [26].

**Table 1:** Basic characteristics of included clinical trials

Study	Design	Main inclusion criteria	Age(years)	*N*/group	Specific study outcomes					
					Definition of remission	Induction week	Maintenance week			AEs
Rosh et al. [13]	Retrospective cohort	Failed IFX treatment	11.1 ± 3.1	115	PCDAI ≤ 10	12		26	52	Infection
Viola et al. [22]	Prospective cohort	Active moderate-to-severe CD, failed IFX treatment	16.1 (9–20)	23	PCDAI ≤ 10	4	12	24	48	Multiple
Russel et al. [23]	Retrospective cohort	Refractory to conventional therapy (thiopurines/steroids/IFX)	14.8 ± 3.1	70	PCDAI ≤ 10	4		26	52	SAE deaths
Hyams et al. [24]	RCT	Moderate to severe CD, failed IFX treatment	13.5 ± 2.47	G1:95 G2:93	PCDAI ≤ 10	4		26	52	Multiple
Cozijnsen et al. [25]	Retrospective cohort	Failed IFX treatment	14 (13–16)	53	PCDAI ≤ 12.5	4	17	34	52	Multiple
Assa et al. [26]	RCT	Biologic-naïve with luminal CD	12.9 ± 2.6	G1:38 G2:40	PCDAI < 10	4	16	24	56	Multiple
Alvisi et al. [2]	Retrospective cohort	Failed IFX treatment	14.8 (9.9–17.1)	44	PCDAI ≤ 10	2		26	52	Multiple
Romeo et al. [27]	Retrospective	Steroid dependency, chronically active disease, steroid resistance	15.1 (13.6–16.0)	36	PCDAI ≤ 10			26	52	
Rinawi et al. [28]	Prospective cohort	Luminal inflammatory CD	13.9 (12.1–15.2)	65	PCDAI ≤ 10	12		24		
Rinawi et al. [28]	Retrospective cohort	Active steroid-dependent CD, failed IFX treatment	14.1 (12.5–15.7)	213	PCDAI < 10	12				Multiple

*RCT* randomized controlled trial, *CD* Crohn’s disease, *IFX* infliximab, *N* number, *G1* group 1, *G2* group 2, *PCDAI* Pediatric Crohn’s Disease Activity Index, *AE* adverse event, *SAE* serious adverse event

The mean or median baseline PCDAI was above 30 in four treatment arms of three studies, signifying moderate to severe disease [22–24]. The mean or median baseline PCDAI was between 10 and 30 in five studies, indicated mild disease on average [2, 13, 26, 28, 29]. Two studies did not report baseline disease severity [25, 27].

The two RCTs comprised four treatment arms, with all patients the randomly assigned at week 4 after induction treatment [24, 26]. In one of these RCTs, the efficacy of different maintenance treatment doses (high doses, 40 mg or 20 mg for body weight ≥ 40 kg or < 40 kg, respectively, and low doses, 20 mg or 10 mg for body weight ≥ 40 kg or < 40 kg, respectively) were investigated [24], whereas the efficacies of proactive (trough concentrations measured at weeks 4 and 8 and then every 8 weeks until week 72) and reactive monitoring (physicians were informed of trough concentrations after loss of response) were compared in the other [26].

The therapeutic dose of ADA varied widely among the included studies. Most subjects received standard adult ADA induction (160/80 mg) via subcutaneous injection, every other week (eow). A maintenance dose of 40 mg was used in 8 of the 12 treatment arms. Furthermore, in one treatment arm, a higher maintenance dose (80 or 40 mg for body weights ≥ 40 kg or < 40 kg eow, respectively) was used, while a lower maintenance dose was used in one of the therapeutic arms in the RCT study described above (20 or 10 mg for body weights ≥ 40 kg or < 40 kg eow, respectively).

The subjects in three studies were IFX-naïve [26–28]. Additionally, participants in two studies had previously received IFX treatment, which was discontinued due to loss of efficacy or related adverse events [2, 25]. In the remaining five studies, some subjects experienced IFX failure prior to ADA therapy [13, 22–24, 29].

### Quality assessment

**Fig. 2:**
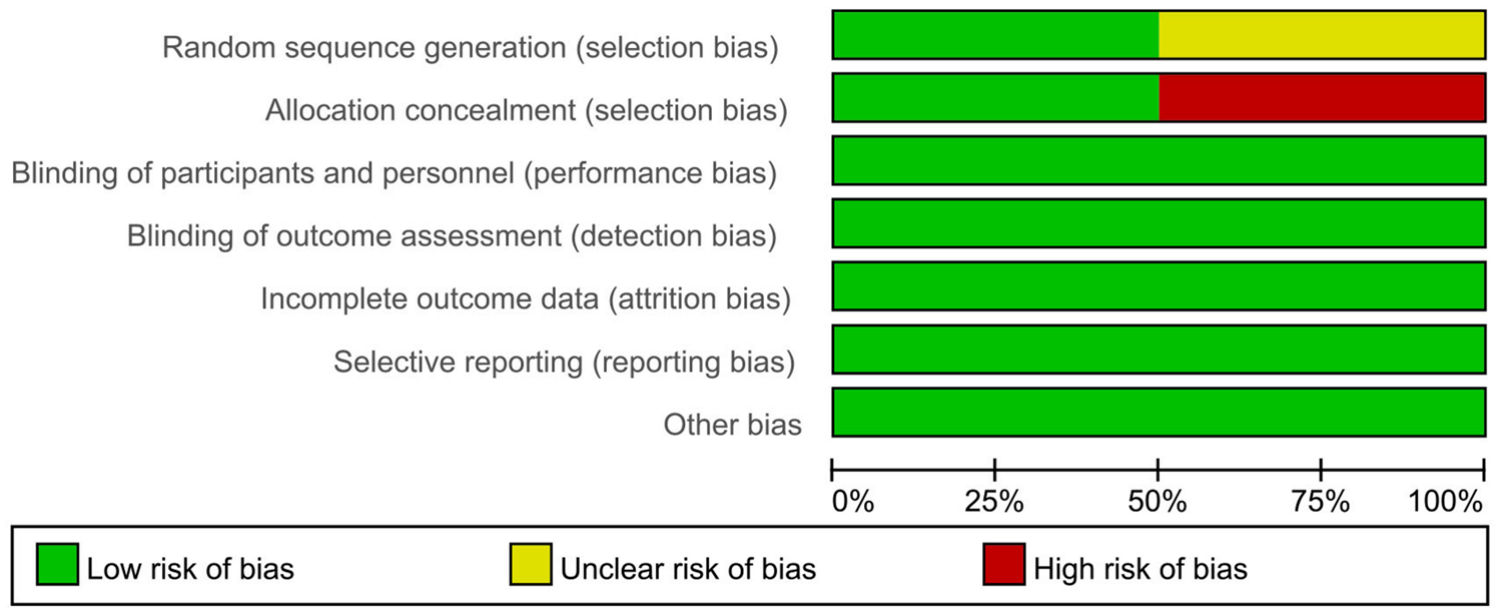
Risk of bias graph (RCTs)

**Fig. 3:**
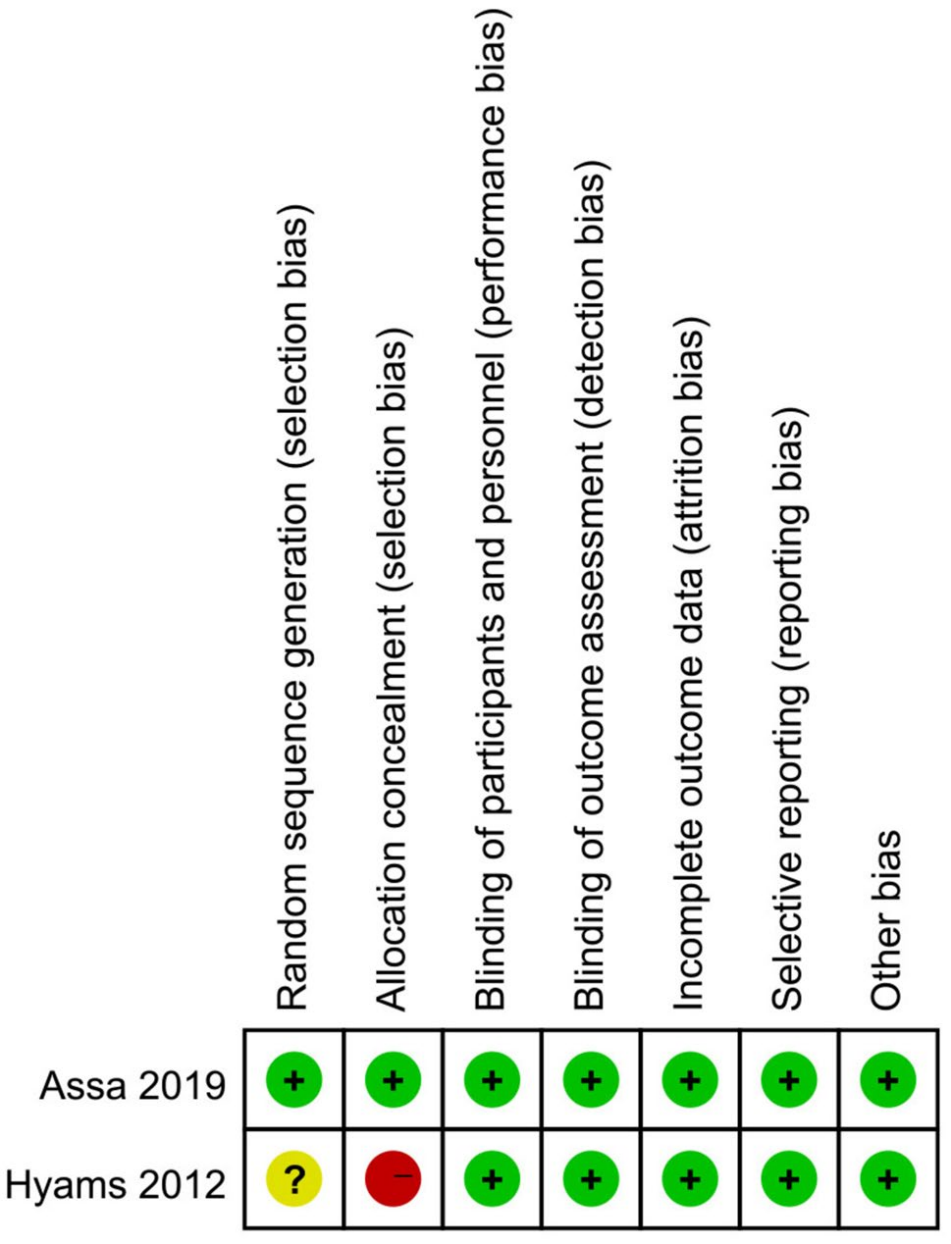
Risk of bias summary (RCTs)

The methodological quality of the two RCTs is summarized in Figs. 2 and 3. One of the RCTs was a phase 3, multicenter, randomized, open-label induction followed by a double-blind maintenance trial, in which subjects were randomly assigned (1:1) high-dose or low-dose ADA. Furthermore, in this study, subjects were stratified according to their week-4 responder status and prior exposure to IFX [24]. The other study was a multicenter RCT, with equal randomization (1:1 ratio), and aims to determine whether proactive therapeutic drug monitoring (TDM) is superior to reactive TDM in children with CD under scheduled monitoring of clinical and biologic measures. Therefore, based on the study design, it was not possible to fully blind the method [26].

Obtaining an objective evaluation of the endpoint was impossible due to the absence of activity comparison in the single-arm studies. In two of the included studies [22, 29], follow-up was less than 1 year, and in another [29], it was only 24 weeks. Furthermore, 12 out of 115 (10.4%) subjects were lost to follow-up in one study [13], and 3 out of 53 (5.6%) were lost to follow-up in another study [25]. The sample size was less than 100 in 6 studies [2, 22, 23, 25, 27, 28]. Regardless, it is acceptable to evaluate items that clearly state the purpose of the research, consistency of the results, methods for the collection of expected data, appropriateness of endpoint indicators to reflect the purpose of the study, and objectivity of the endpoint evaluation. As shown in Table 2, the quality of clinical trials ranged from moderate to high.

**Table 2:** Assessment of study quality (single-arm studies)

Study	The purpose of the research is clearly stated	Consistency of enrolled patients	Collection of expected data	Endpoints that appropriately reflect the purpose of the study	The objectivity of endpoint evaluation	Adequate follow-up time	Loss to follow-up rate is less than 5%	Whether the sample size was estimated	total
Rosh et al. [13]	2	2	2	2	0	2	0	2	12
Viola et al. [22]	2	2	2	2	0	1	2	1	12
Russel et al. [23]	2	2	2	2	0	2	2	1	13
Cozijnsen et al. [25]	2	2	2	2	0	2	1	1	12
Alvisi et al. [2]	2	2	2	2	0	2	2	1	13
Romeo et al. [27]	2	2	2	2	0	2	2	1	13
Rinawi et al. [28]	2	2	2	2	0	0	2	1	11
Rinawi et al. [28]	2	2	2	2	0	2	2	2	14

Funnel plots were used to assess the potential publication bias in the reporting of remission maintenance (Fig. S2). The pooled results showed no evidence of a significant publication bias. Additionally, the Egger test was performed to evaluate the publication bias in the reporting remission maintenance. We obtained *P* = 0.633, which corroborated no significant publication bias (Fig. S3).

### Outcomes of interest

**Fig. 4:**
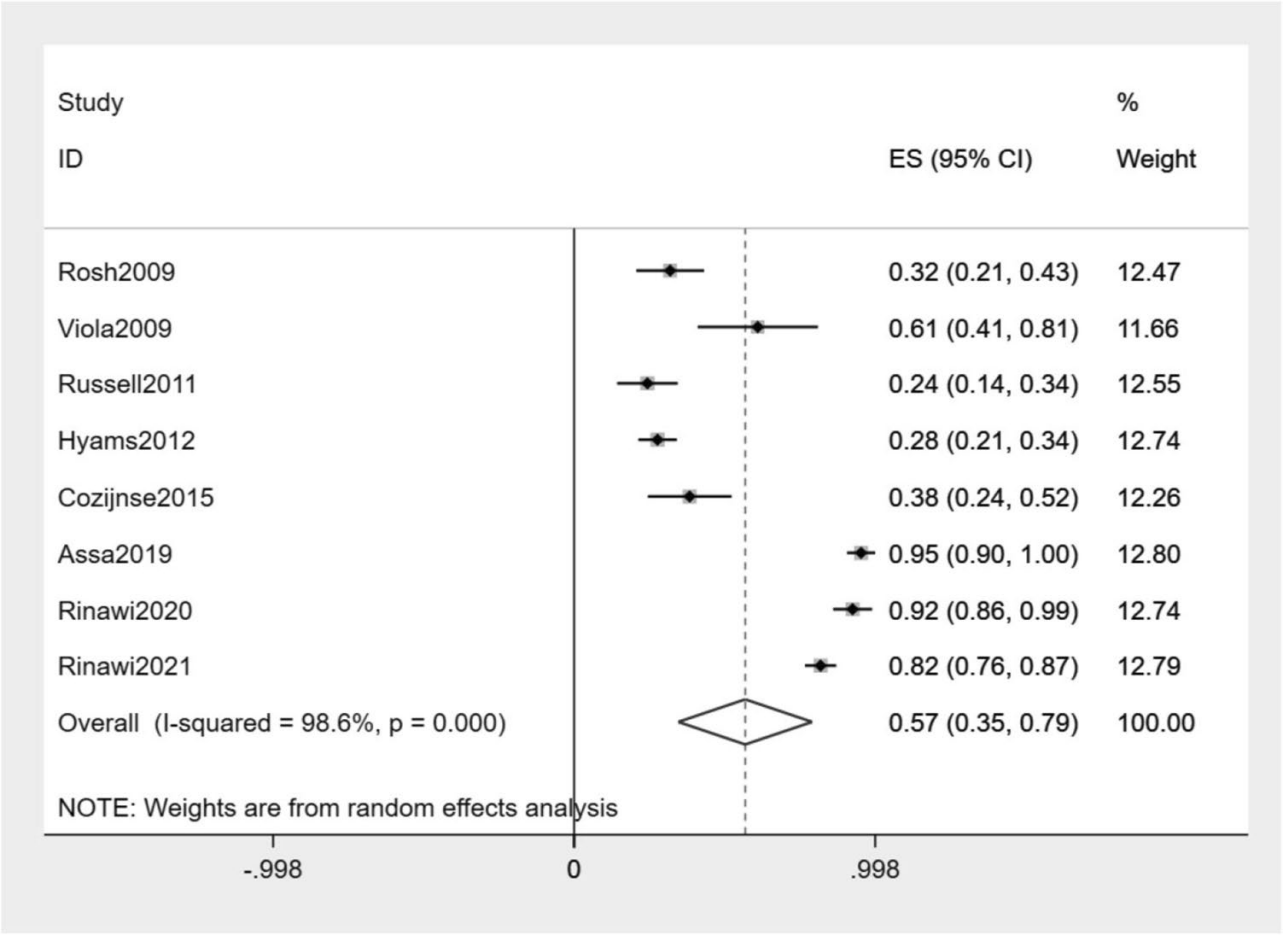
Forest plot of induction of remission

**Fig. 5:**
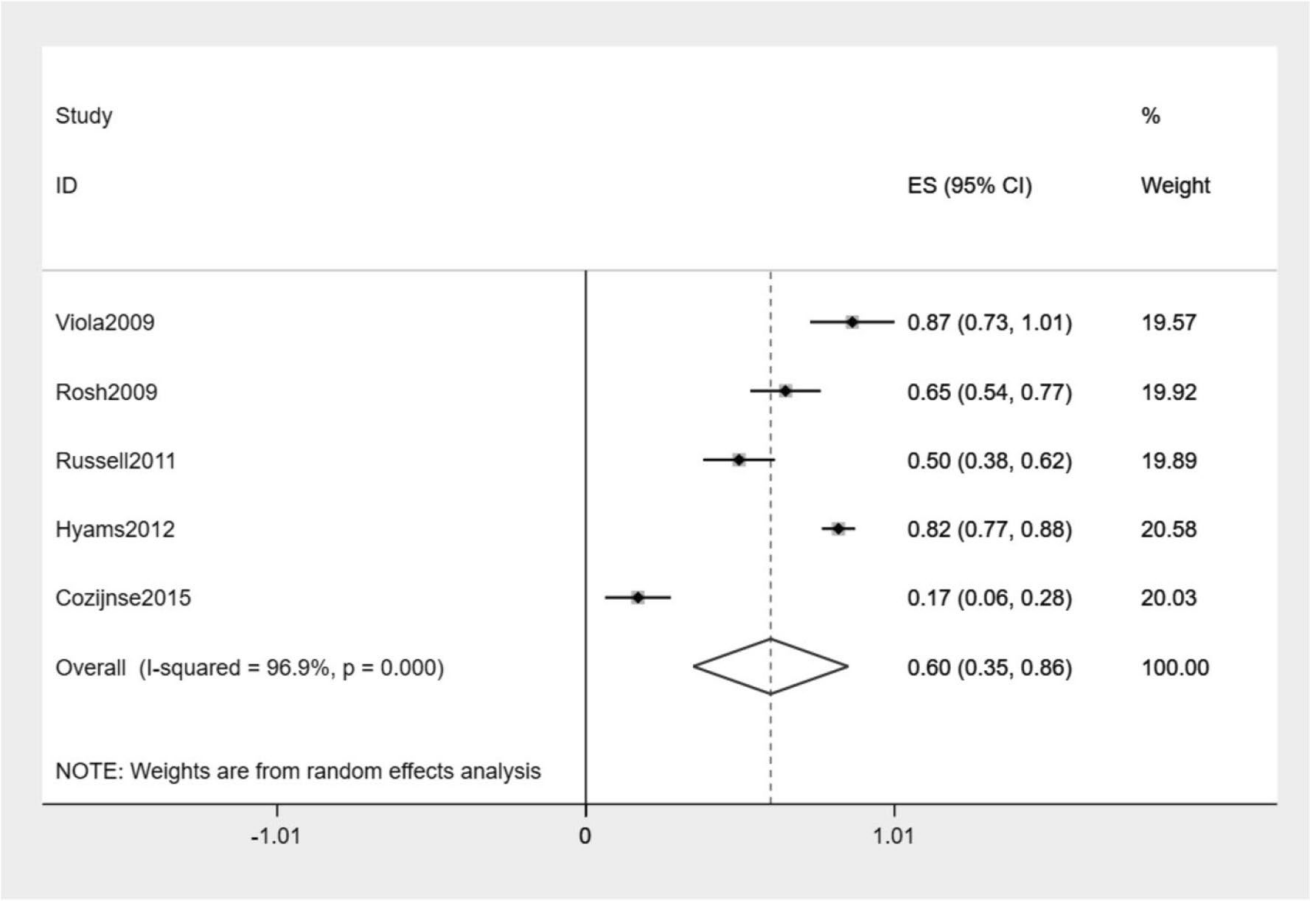
Forest plot of induction of response

**Fig. 6:**
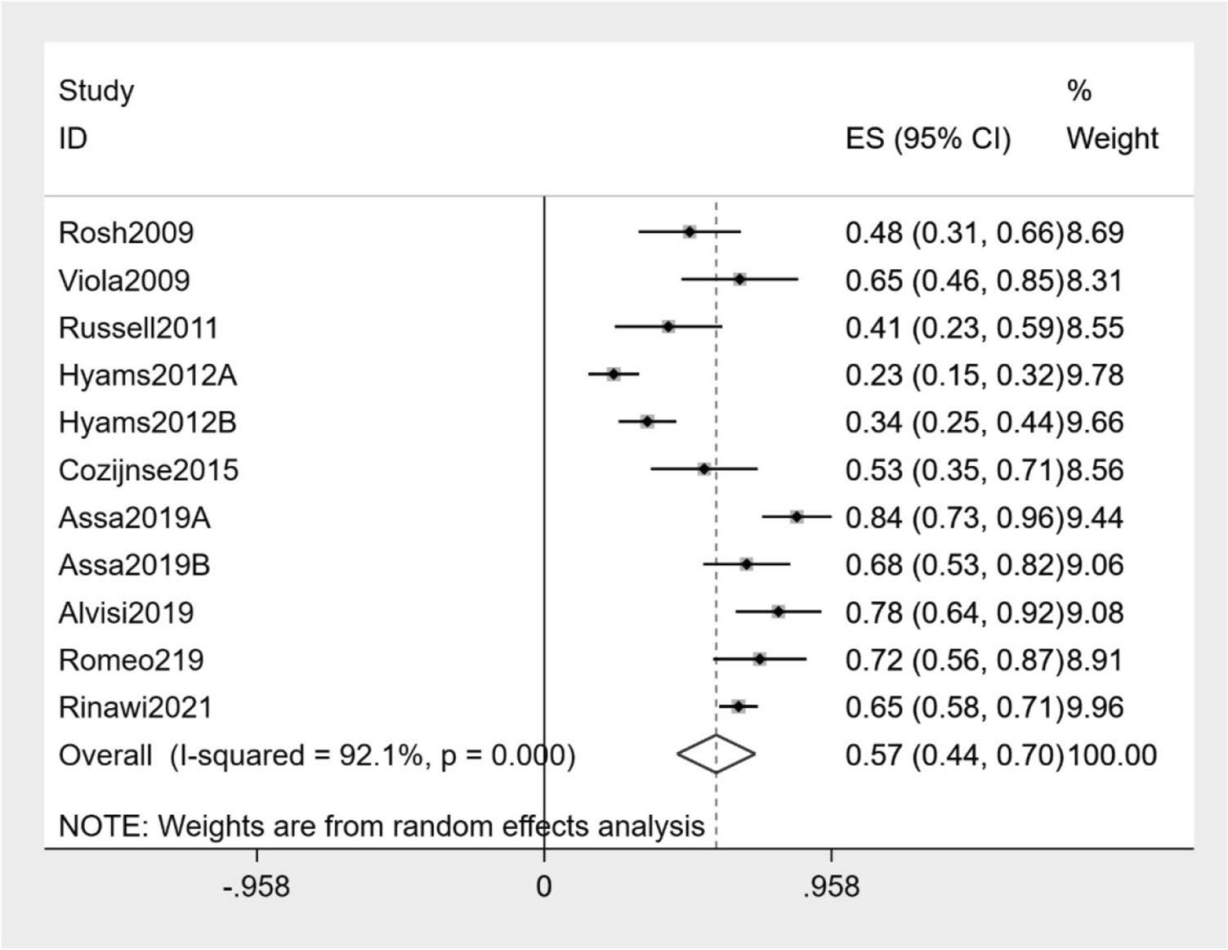
Forest plot of maintenance of remission

**Fig. 7:**
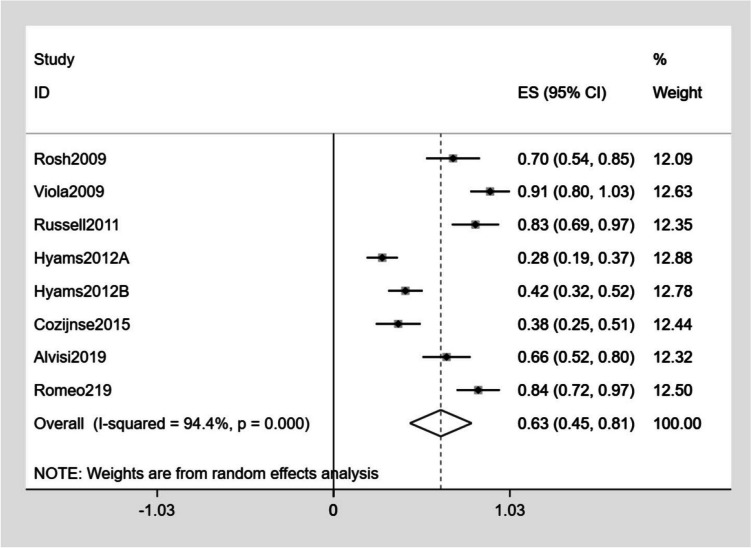
Forest plot of maintenance of response

The detailed outcomes of each study are presented in Table 3. The calculation of the pooled weighted proportions indicated that 59% (95% CI 25–61%) of the participants achieved induction of remission, 60% (95% CI 6–35%) achieved induction of response, 57% (95% CI 55–79%) achieved maintenance of remission, and 63% (95% CI 30–87%) achieved maintenance of response (Figs. 4, 5, 6, 7).

**Table 3:** The detailed outcomes at the end of treatment

Study	Group	Base line PCDAI	Induction of clinical remission No. (%)	Induction of clinical response No. (%)	Maintenance of remission No. (%)	Maintenance of response No. (%)	maintenance dose	Injection-related reactions No. (%)	Infection No. (%)	SAE No. (%)	Discontinuation	Deaths No. (%)
Rosh et al. [13]		25 ± 15	21 (31.8)	43 (65.1)	16 (48.4)	23 (69.7)	40 mg		2 (1.7)		12 (10.4)	
Viola et al. [22]		36.5 ± 5.7	14 (60.8)	23 (86.9)	15 (65.2)	21 (91.3)	80 mg (≥ 40 kg) 40 mg (< 40 kg)	4 (17.4)	2 (8.7)		0	
Russel et al. [23]		37.5	17 (24.2)	35 (50.0)	12 (41.3)	12 (41.3)	40 mg		6 (8.6)	4 (5.7)	0	2 (2.9)
Hyams et al. [24]	G1 G2	> 30 > 30	NR NR	NR NR	22 (23.2) 32 (34.4)	27 (28.4) 39 (41.9)	40 mg (≥ 40 kg) 20 mg (≥ 40 kg)	10 (10.5) 9 (9.7)	47 (49.5) 56 (60.2)	11 (11.6) 19 (20.4)	12 (12.6) 15 (16.1)	
Cozijnsen et al. [25]		NR	18 (38.2)	8 (17.0)	16 (53.3)	4 (13.3)	40–80 mg (≥ 40 kg) 20–40 mg (< 40 kg)	5 (9.4)	14 (26.4)	1 (1.9)	3 (5.6)	
Assa et al. [26]	G1 G2	A:18.3 B:17.5	34 (89.4) 35 (87.5)	NR NR	32 (84.2) 27 (67.5)	NR NR	40 mg 40 mg		1 (2.6) 2 (5)	4 (10.5) 4 (10)	2 (5.2) 0	
Alvisi et al. [2]		24.94	NR	NR	25 (78.1)	4 (12.5)	40 mg	1 (2.2)		2 (4.5)	2 (4.5)	
Romeo et al. [27]		NR	NR	NR	23 (71.9)	27 (84.3)	NR				0	
Rinawi et al. [28]		17.5	60 (92.3)	NR	57 (87.7)	NR	40 mg (≥ 40 kg) 20 mg (< 40 kg)					
Rinawi et al. [28]		17.5	174 (81.7)	NR	138 (64.8)	NR	40 mg	10 (4.7)	4 (1.9)	10 (4.7)	10 (4.6)	

*No* number, *NR* no report, *PCDAI* Pediatric Crohn’s Disease Activity Index, *AE* adverse event, *SAE* serious adverse event, *G1* group 1, *G2* group 2, *NR* no report

The most frequently reported adverse event was infection. A total of 134 (15.1%) patients developed infections. Furthermore, injection-related reactions were reported in 39 patients (4.4%) and 45 (5%) SAEs occurred in 885 patients. The most frequently reported SAEs were serious infections (30 patients); other SAEs included a meningitis secondary to a sinusitis (1 patients), pancreatitis (1 patients), severe neurological symptoms (3 patients), severe psoriasis (5 patients), and severe vasculitis (3 patients). Furthermore, two deaths were reported in one study. Other common adverse effects included neurological symptoms, neutropenia, vasculitis, and allergic and psoriasiform skin lesions.

### Sensitivity and subgroup analysis

The calculation of the pooled weighted proportions indicated high heterogeneity among studies. Thus, further sensitivity and subgroup analyses were conducted to investigate important confounding factors (sensitivity analysis, see Fig. S4). The results of the subgroup analysis according to the baseline PCDAI score, study design, prior IFX exposure, and maintenance dose are presented in Fig. S5.

### Induction of remission

In the sensitivity analysis of remission induction, excluding any single arm did not significantly alter the heterogeneity among the studies. The subgroup analysis results also suggested that the proportion of patients with remission induction was significantly higher in children with PCDAI < 30 at baseline than in those with ≥ 30 (0.76 [0. 57–0.95] vs. 0.34 [0.20–0.49]). Furthermore, subgroup analysis in terms of whether the patients had previously received IFX treatment revealed no significant difference between the subgroup of IFX-exposed patients and the subgroup of some subjects without prior IFX treatment (0.38 [0. 24–0.52] vs. 0.45 [0.17–0.74]). However, ADA was significantly effective in the subgroup of IFX-naïve patients (0.94 [0.90–0.98]), suggesting a possible risk of bias.

### Maintenance of remission

In the sensitivity analysis of remission maintenance, we noted that excluding any single study did not significantly change the heterogeneity among the studies. Subgroup analysis further revealed that children with PCDAI < 30 at baseline showed a significantly higher proportion of maintenance of remission than those with score ≥ 30 (0.69 [0. 59–0.80] vs. 0.39 [0.24–0.54]). Additionally, whether the patients were from non-RCTs or RCTs resulted in no significant difference in the subgroup analysis results (0.61 [0.53–0.70] vs. 0.52 [0.24–0.80]). Moreover, there was no significant difference in the proportion of patients in the 40 mg doses cohort and ≥ 40 mg arm (0.58 [0.42–0.74] vs. 0.65 [0.46–0.85]); however, the < 40 mg arm achieved a lower proportion of maintenance remission 0.34 (0.25–0.44). Our results also indicated no significant difference between the subgroup in which all patients experienced IFX failure and the subgroup in which some patients experienced IFX failure (0.66 [0. 42–0.91] vs. 0.46 [0.29–0.63]). However, ADA was significantly more effective in the subgroup with IFX-naïve patients (0.75 [0.65–0.86]).

### Maintenance of response

In the sensitivity analysis, excluding any single study did not significantly alter the heterogeneity among the studies. Additionally subgroup analysis revealed no significant difference between the two cohorts with PCDAI scores > 30 and < 30 at baseline (0.61 [0. 31–0.91] vs. 0.68 [0. 0.57–0.78]). We also noted that the rate of maintenance response was greater in non-RCTs than RCTs (0.72 [0.56–0.88] vs. 0.35 [0.22–0.48]). In the ADA dose subgroup analysis, we noted that the response rate increased in a dose-dependent manner (0.42 [0.32–0.52] vs. 0.57 [0.35–0.78] vs. 0.91 [0.80–1.03]) for < 40, 40, and ≥ 40 mg, respectively). There was no significant difference between the subgroup in which some patients experienced IFX failure and the subgroup in which all patients prior to IFX treatment (0.63 [0.37–0.88] vs. 0.52 [0.24–0.79]). However, ADA was significantly more effective in the IFX-naïve subgroup (0.84 [0.72–0.97]).

## Discussion

Biological therapies, e.g., anti-TNF-α agents, have been extensively used in pediatric CD because they have been demonstrated to positively modify the natural history of IBD and facilitate mucosal healing. The efficacy of ADA in achieving clinical remission in children with CD has also been demonstrated in various studies [13, 23, 24]. However, different studies with different therapeutic doses, follow-up periods, and sample sizes have used different criteria to assess disease severity, limiting the generalization of results in clinical practice and the possibility of comparisons among them. In summary, we identified 10 clinical trials, with 885 subjects enrolled, that met the inclusion criteria. The major finding of our study was that the pooled remission and response rates of ADA induction and maintenance were > 50%, suggesting that ADA is effective as a treatment for children with CD. Adverse events varied between the included studies, but were usually not severe.

In clinical practice, one of the therapeutic goals of a new agent is the rapid induction of response or remission. Data over the years corresponding to adults shows that ADA exerts long-term clinical benefits [30]. However, a significant number of children and adults lose response to ADA over time, and thus, require either a dose increase or reduction in the dosing interval [13, 31]. Only a few published studies have reported an optimal ADA dose for pediatric patients with CD [24], either for the induction or maintenance of remission. Therefore, the doses used in pediatric practice are extrapolated from relevant adult studies [9, 32] and pediatric rheumatology studies [33]. Therefore, it is difficult to conclude on an optimal dose CD treatment in children. In the current study, we noted that most of the subjects in the included studies received a standard adult ADA induction dose of 160/80 mg, while the 40-mg dose was frequently used in maintenance therapy. Subgroup analysis showed no significant difference in the overall response rates between the 80 mg and 40 mg maintenance groups. However, the remission rate in the 20 mg group was significantly lower than that in the 40 mg group. Subgroup analysis further suggested that a dose of 40 mg may be the most effective in maintaining clinical remission and clinical response.

ADA effectively maintains a long-term response and remission in children with CD. In the current study, the follow-up period varied between 48 and 208 weeks, with one study reporting a maximum follow-up period of only 24 weeks [28]. We also observed maintenance remission rates of 60%, 57%, and 62% at weeks 26, 52, and 104, respectively.

Patients with CD previously exposed to TNF-α were more likely to exhibit a refractory phenotype [34, 35]. Subgroup analysis in this study demonstrated that the efficacy of ADA was higher in TNF-α-naïve patients than in their TNF-α-exposed counterparts. This is consistent with the results of Song et al. [16]. However, Yin et al. [17] assessed the efficacy and safety of ADA in inducing and maintaining remission of participants with CD; their study included four RCTs, and based on their observations, they concluded that efficacy rates were similar between the TNF-α-naïve and TNF-α-exposed subgroups. The most important reason to alter the efficacy of a second anti-TNF in CD patients will depend on the cause for switching. The remission rate will be higher when the reason for discontinuing the first anti-TNF is intolerance rather than secondary or primary failure. Probably, this explains why Yin et al. concluded that efficacy rates were similar between the TNF-α-naïve and TNF-α-exposed subgroups [11]. However, these findings need to be interpreted cautiously owing to several limitations, including differences between the included studies in terms of study design, differences in treatment periods, and the use of different doses of ADA administered at various intervals throughout the studies periods, irrespective of concomitant therapy. Therefore, large-scale prospective clinical trials are required to validate these findings.

Regarding side effects, only two deaths were reported in one study [23], and they were due to central venous catheter sepsis, which resulted in septic shock. Although there are recognized morbidity and mortality rates in adult clinical studies, the reported combined mortality rate in clinical trials of ADA is not higher than the overall value expected for patients with CD [36]. However, these data need to be interpreted cautiously, as patients in clinical trials might not represent those seen in clinical practice. Moreover, follow-up might not be sufficiently long for some serious events, such as malignancy, to occur.

To the best of our knowledge, this is the first meta-analysis to evaluate the efficacy and safety of ADA in children with CD. However, this study had some limitations. First, differences in study design, baseline disease severity, and treatment dose may have contributed to heterogeneity in the meta-analysis outcomes. Second, the RCTs are considered the most scientifically rigorous study design for evaluating the effectiveness of interventions [37]. Considering the higher severity of the disease in pediatric patients and the fact that CD influences the growth and development of children as a special group, parents are often reluctant to provide consent for their children to be included in such trials. One of the RCTs included in this study involved the comparison of different ADA dose, while the other involved the comparison of proactive and reactive monitoring, no placebo used. Third, in most of the clinical trials, the length and adequacy of follow-up were unclear. Furthermore, most of the included studies had the limitation of a small sample size: only three studies had sample sizes above 100. Fourth, data on endoscopic outcomes were unavailable. Finally, cost analysis was not performed in this systematic review and meta-analysis owing to insufficient data.

## Conclusion

Our meta-analysis showed that ADA provides significant benefits to children with CD. However, the available literature is limited by the risk of bias and small sample size. Therefore, further prospective studies are required to confirm the efficacy and safety of ADA in pediatric patients with CD.

### Supplementary Information

Below is the link to the electronic supplementary material.

Supplementary file1 (DOCX 1648 KB)

## Data Availability

The datasets generated during and/or analyzed during the current study are available from the corresponding authors upon reasonable request.
