# Supplementary material for: Efficacy and safety of adalimumab in pediatric patients with Crohn’s disease: A systematic review and meta-analysis
Source: Eur J Clin Pharmacol. 2023 Dec 29;80(3):395–407. doi: 10.1007/s00228-023-03613-1 (PMC10873464; doi:10.1007/s00228-023-03613-1)

**Fig. S1.** PubMed search history

Search history on January 6, 2023

Search history on November 3, 2023

**Fig. S2.** Funnel plots of maintenance of remission


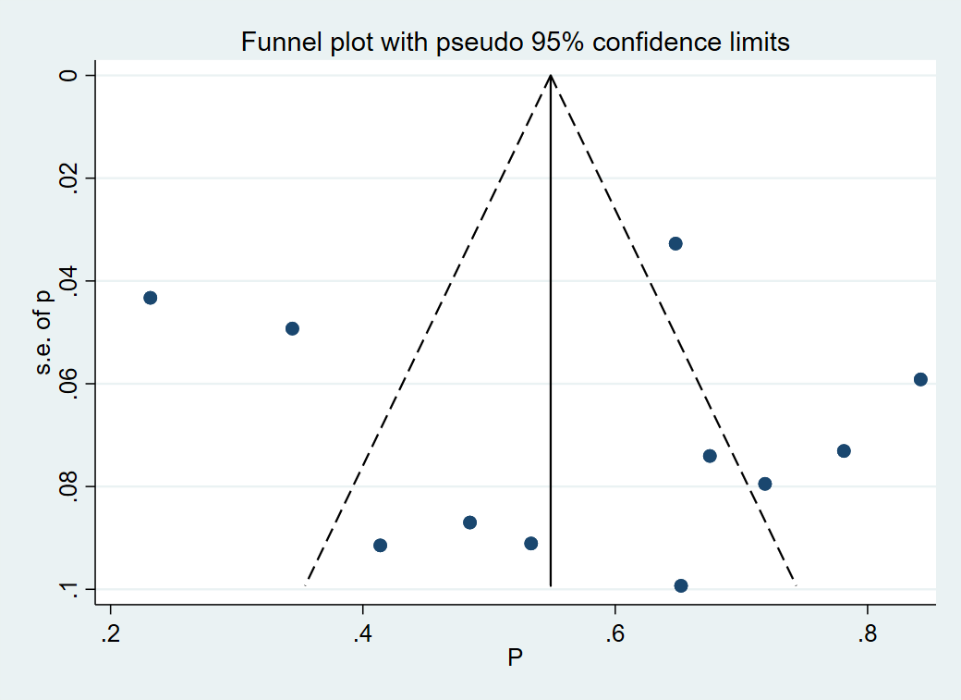


**Fig. S3.** egger test of publication offset of remission


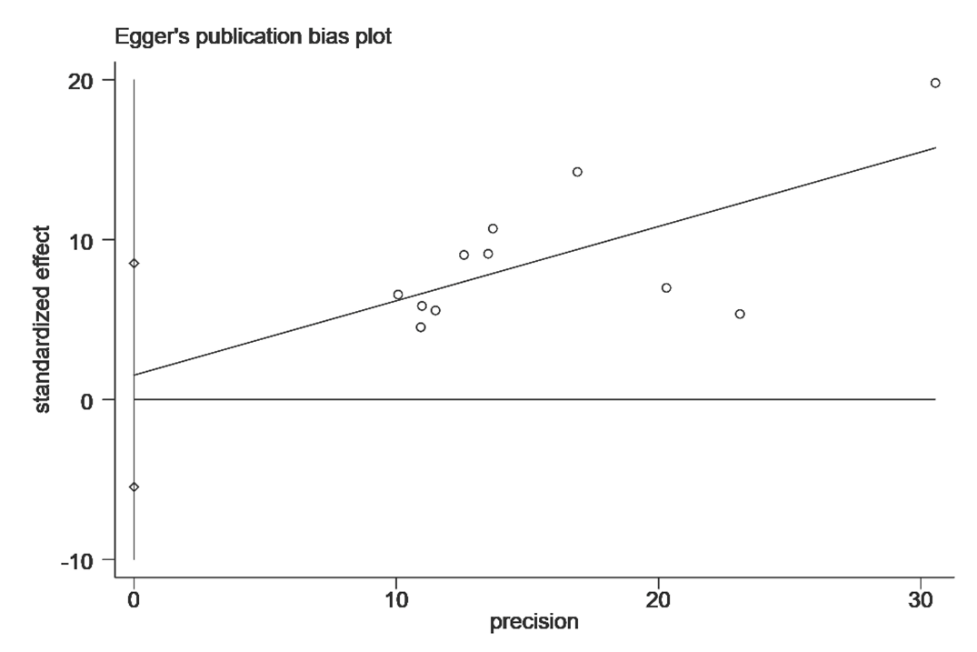


**Fig. S4.** sensitivity analysis of (A) induction of remission; (B) induction of response;（C）maintenance of remission; (D) maintenance of response.

**A**


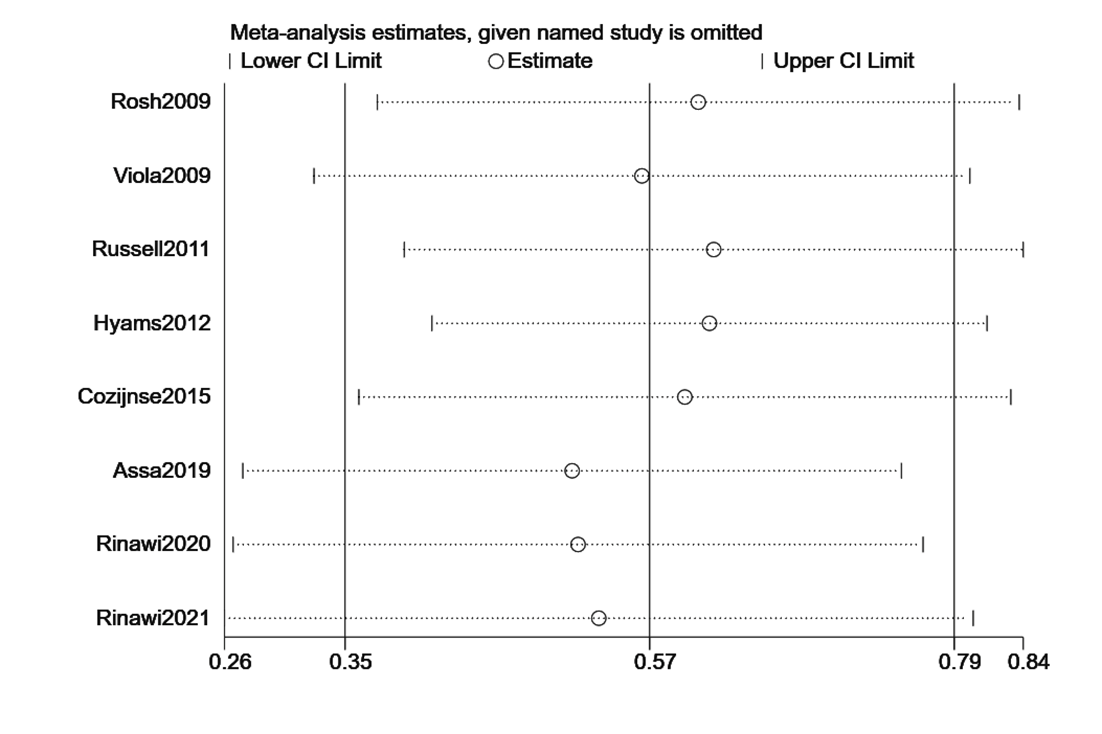


**B**

**
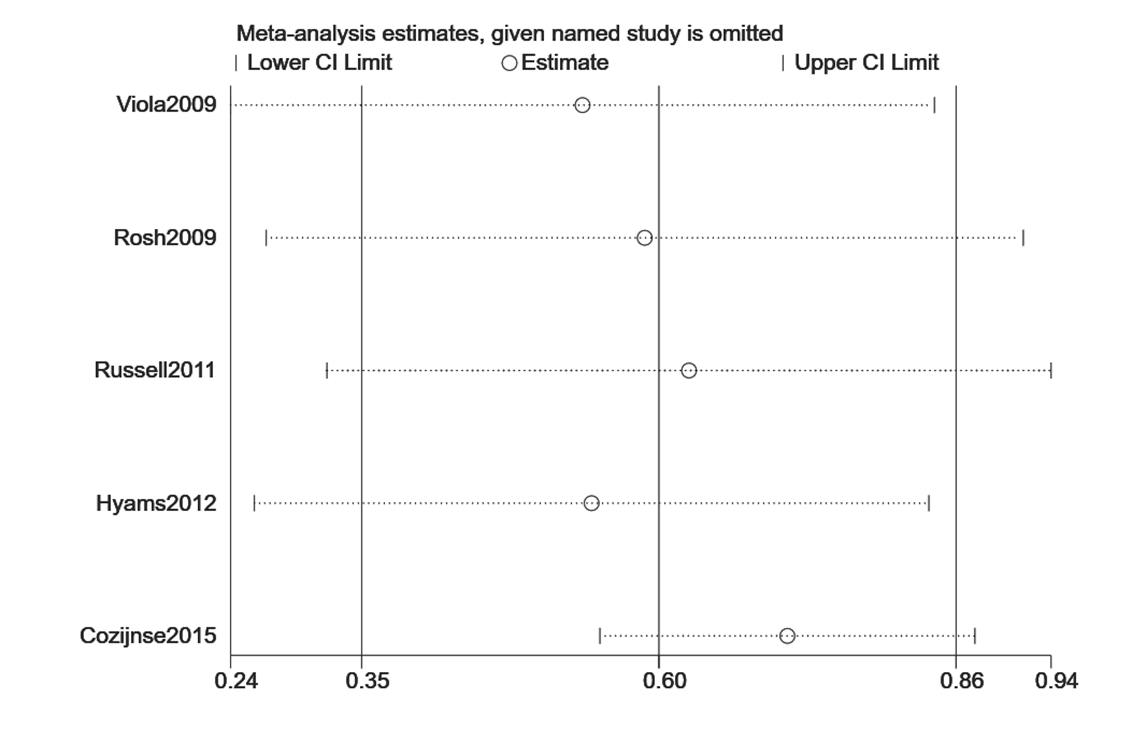
**

**C**

**
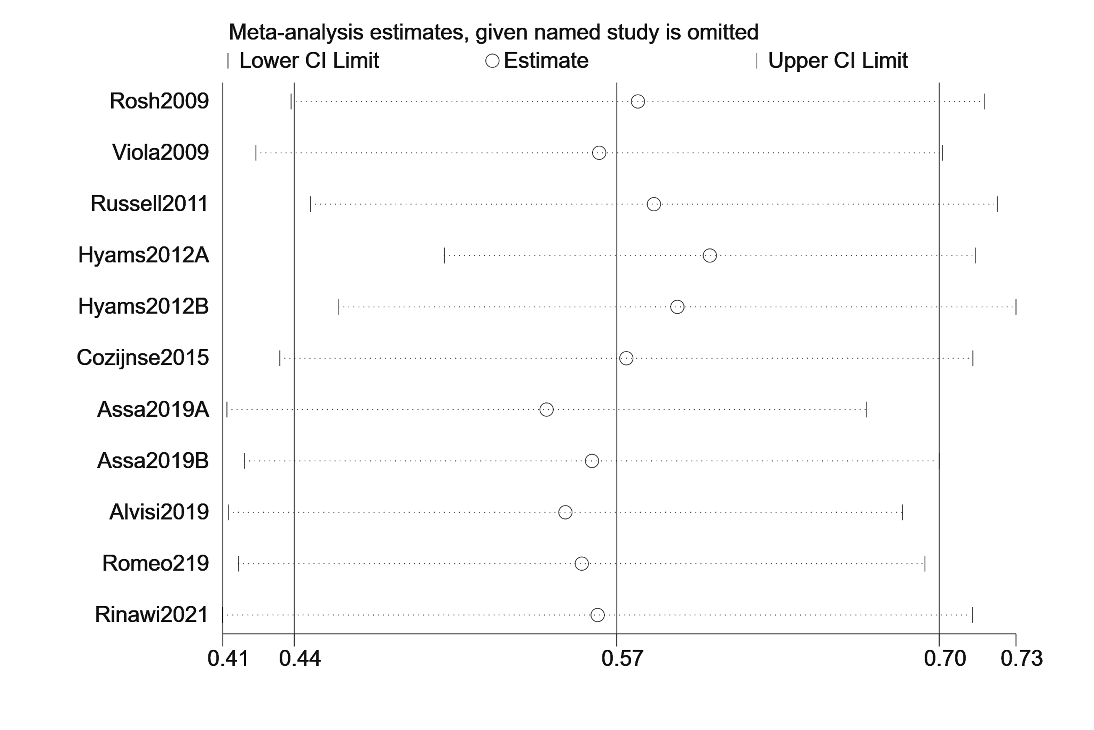
**

**D**

**
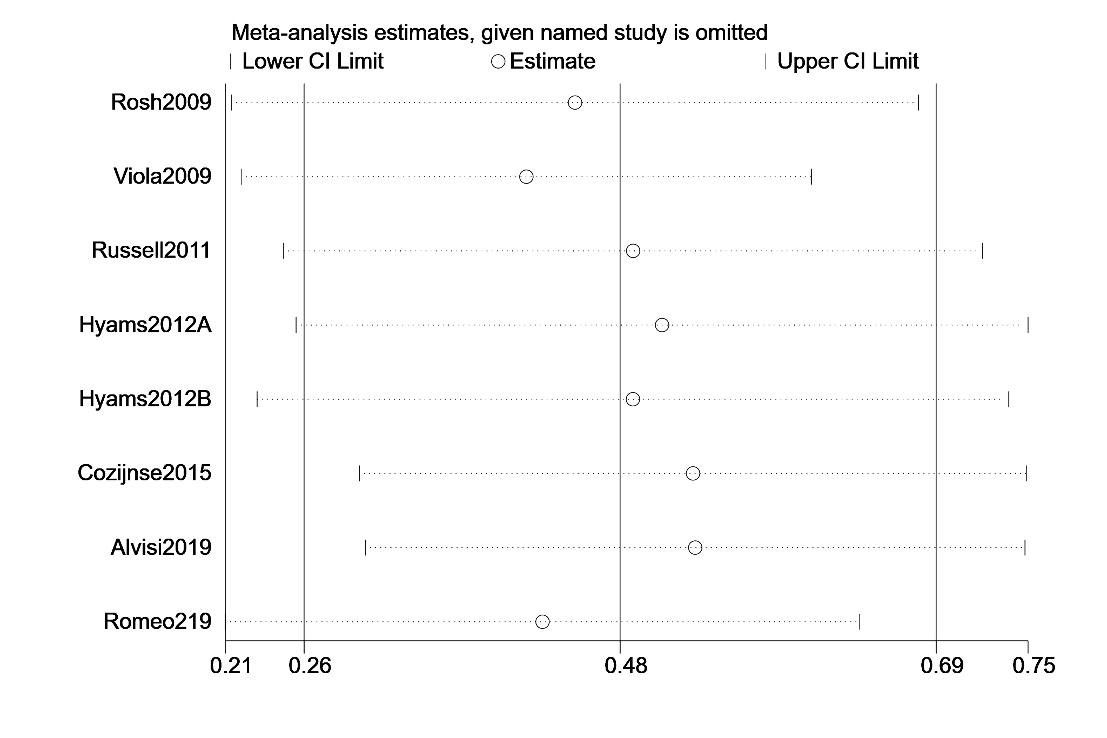
**

**Fig. S5.** Results of the subgroup analysis (A) Forest plot: results of the subgroup analysis by basic PCDAI of subjects; (B) Forest plot: results of the subgroup analysis by types of studies; (C) Forest plot: results of the subgroup analysis by maintenance dose; (D) Forest plot: results of the subgroup analysis by IFX-exposed.

1. Forest plot: results of the subgroup analysis by basic PCDAI of subjects

1.Induction of remission


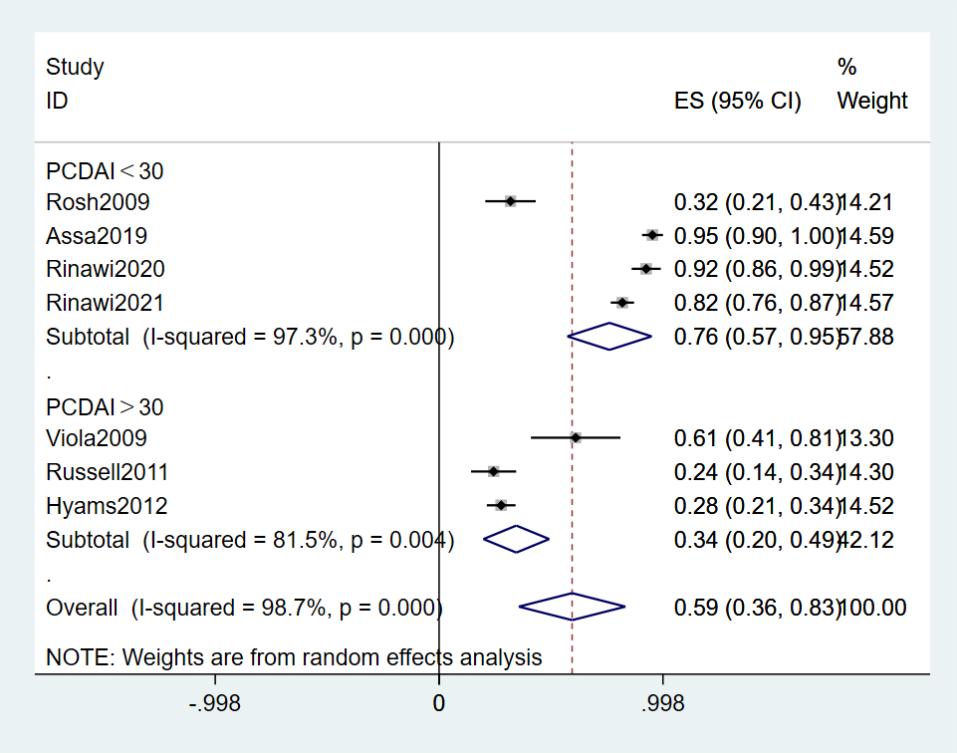


2. Maintenance of remission


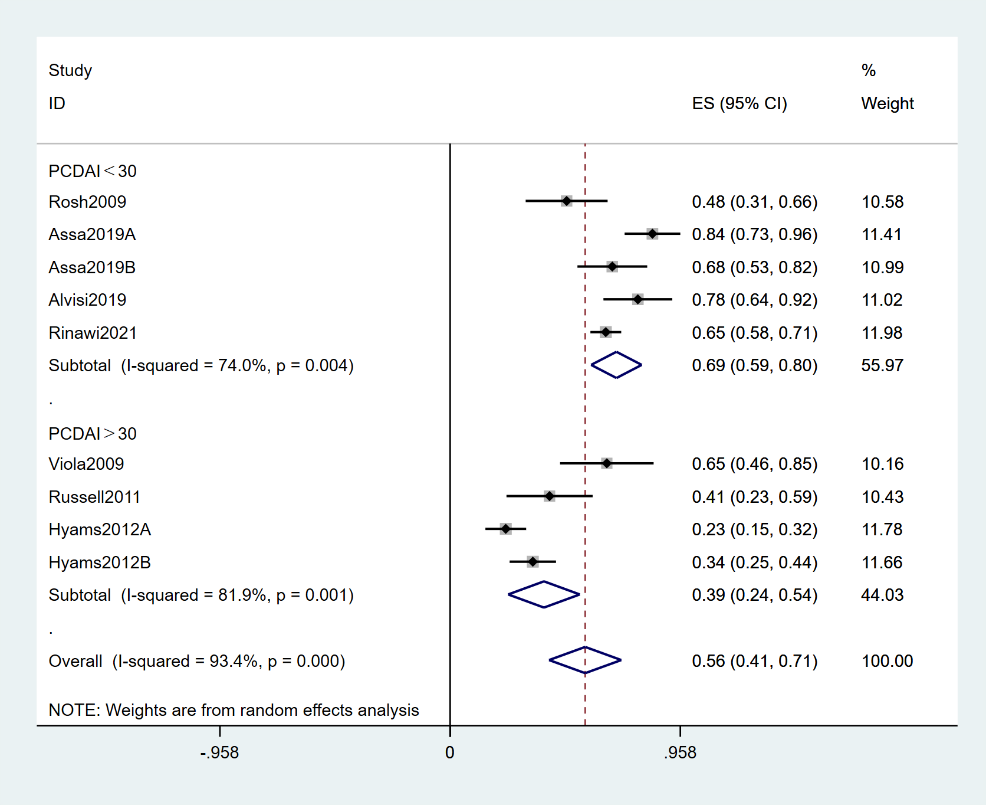


3. Maintenance of response


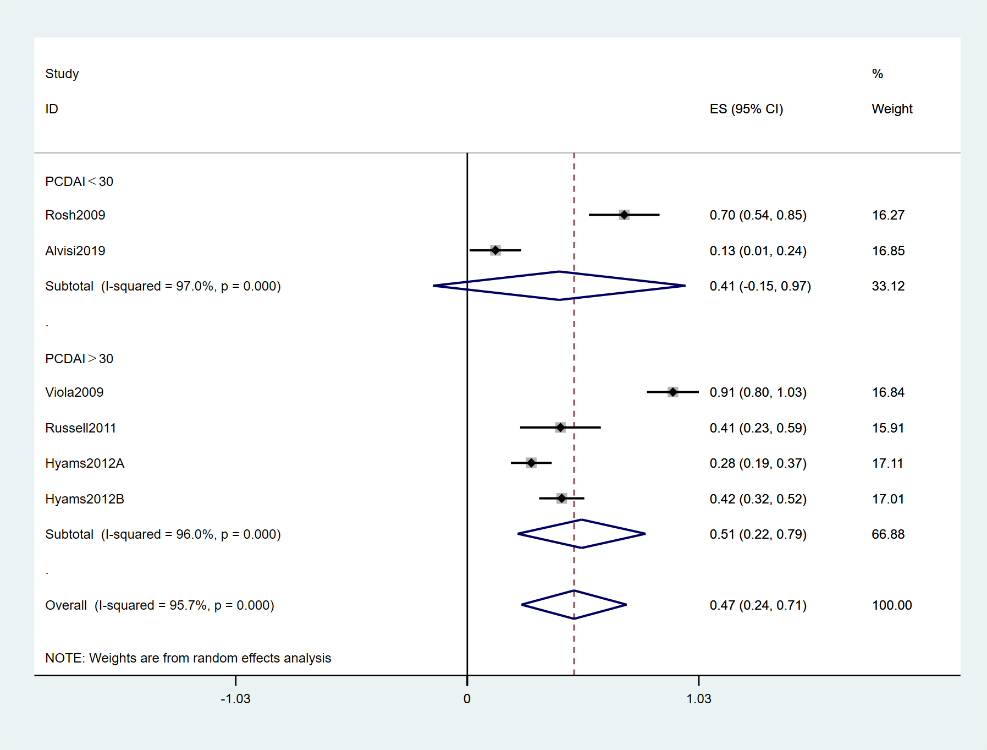


1. Forest plot: results of the subgroup analysis by types of studies.

1. Maintenance of remission


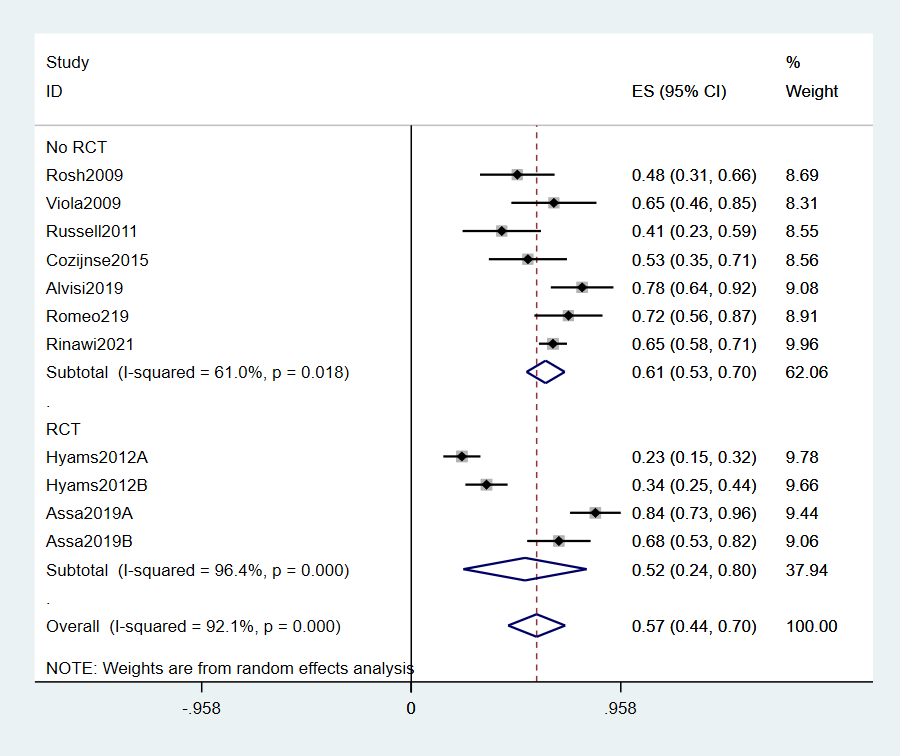


2. Maintenance of response


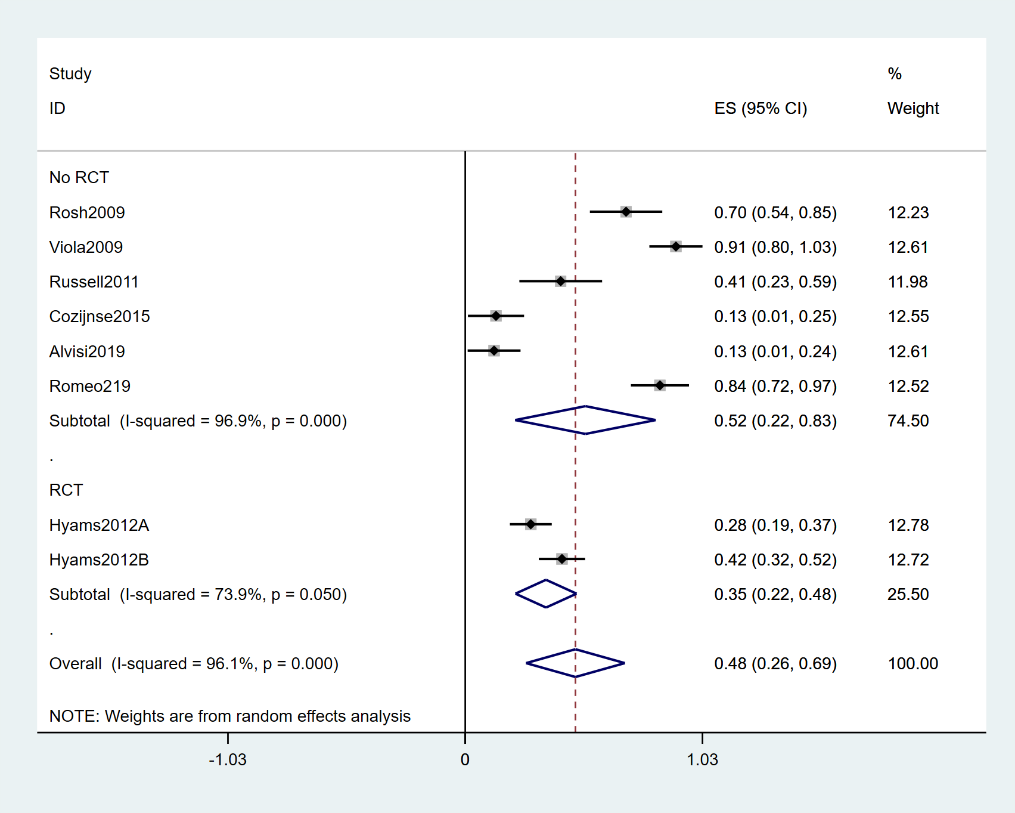


1. Forest plot: results of the subgroup analysis by maintenance dose.

1. Maintenance of remission


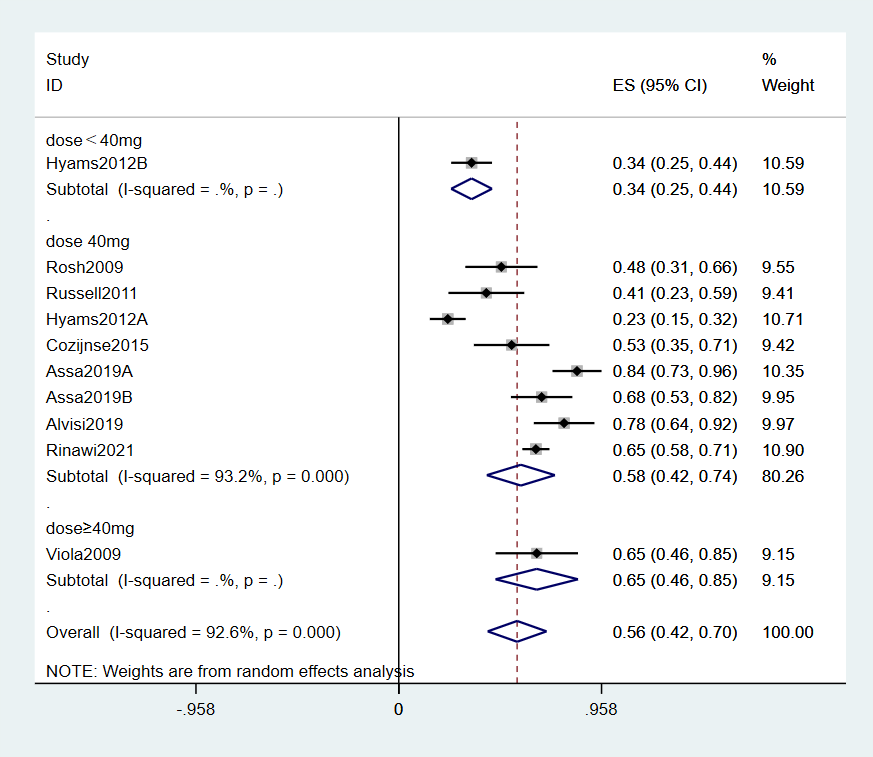


2. Maintenance of response


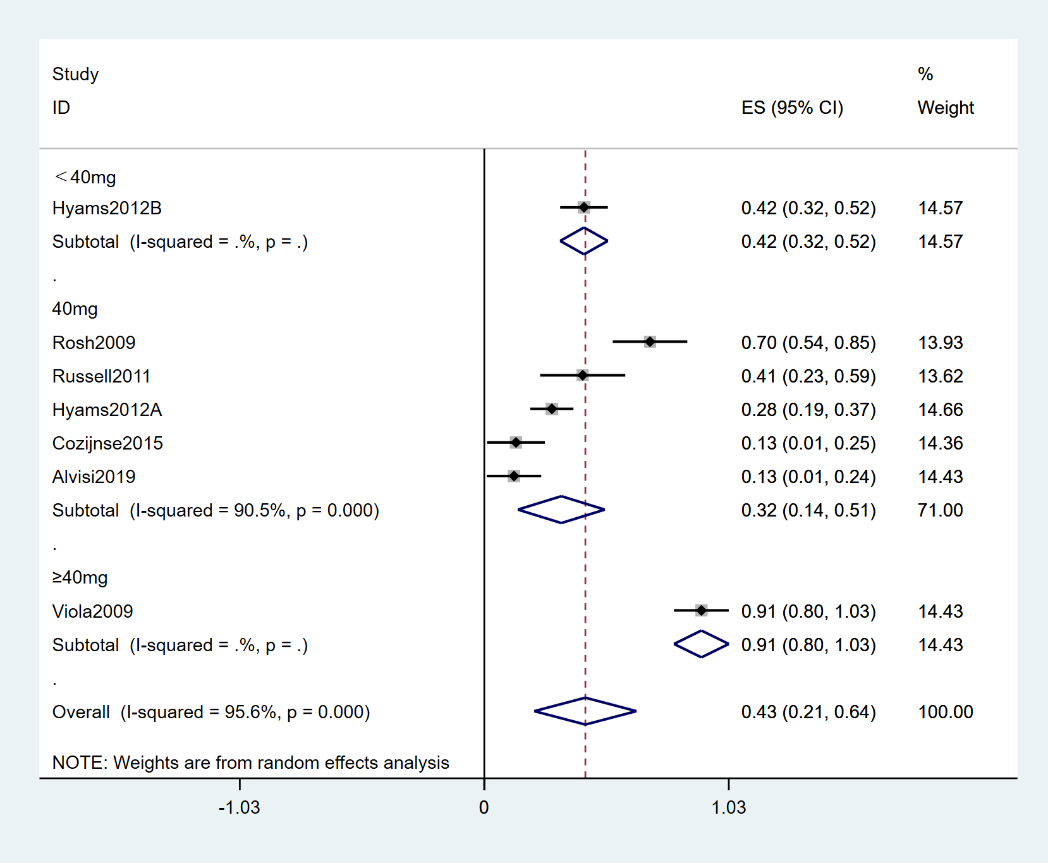


(D) Forest plot: results of the subgroup analysis by IFX-exposed.

1.Induction of remission


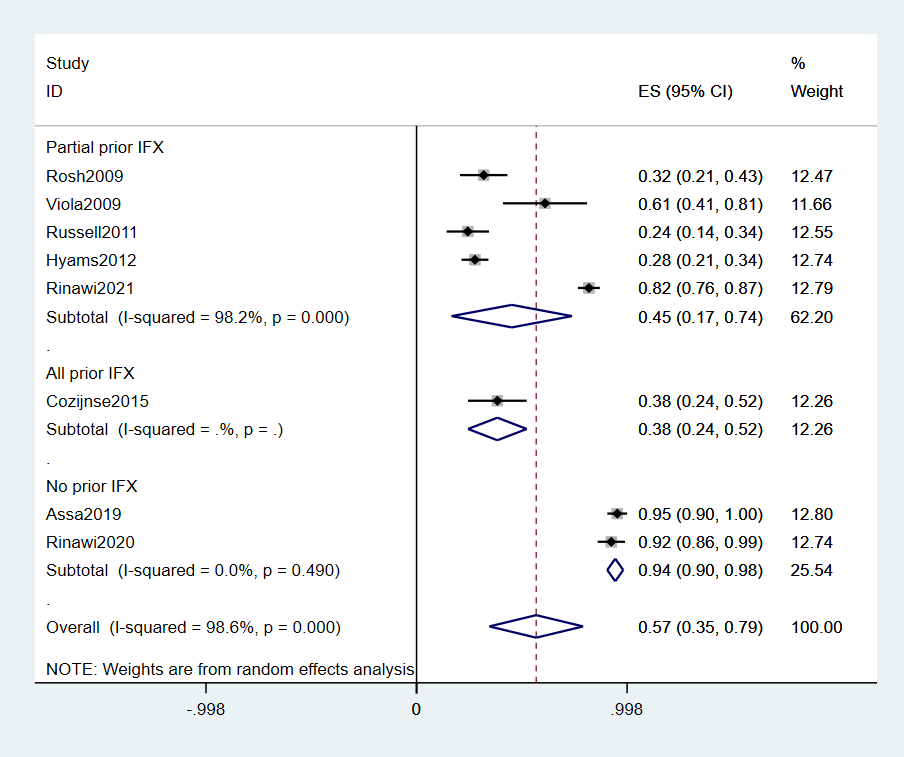


2. Maintenance of remission


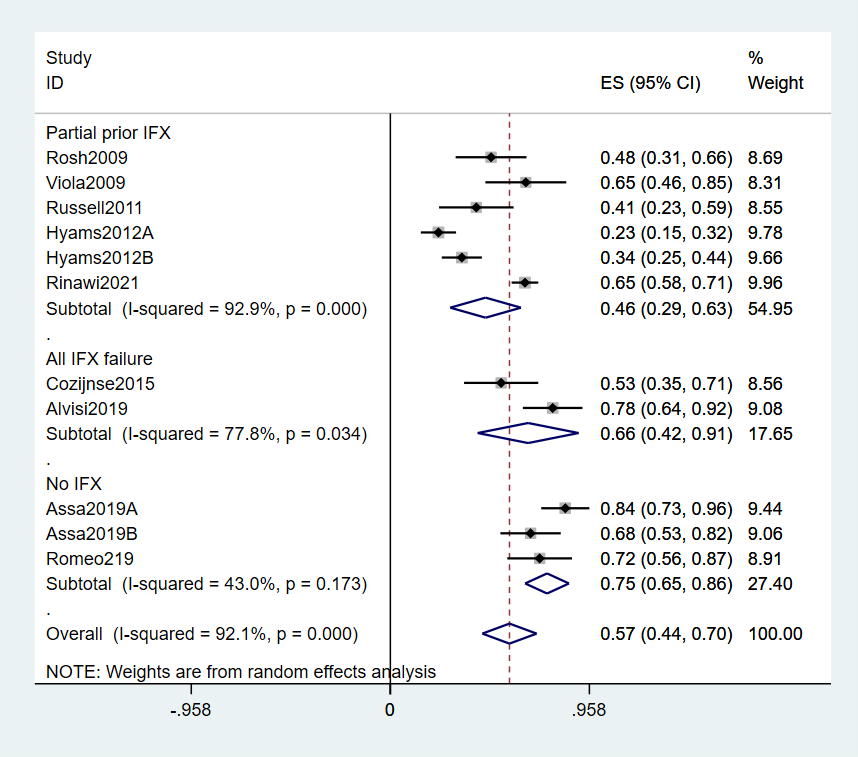


3. Maintenance of response


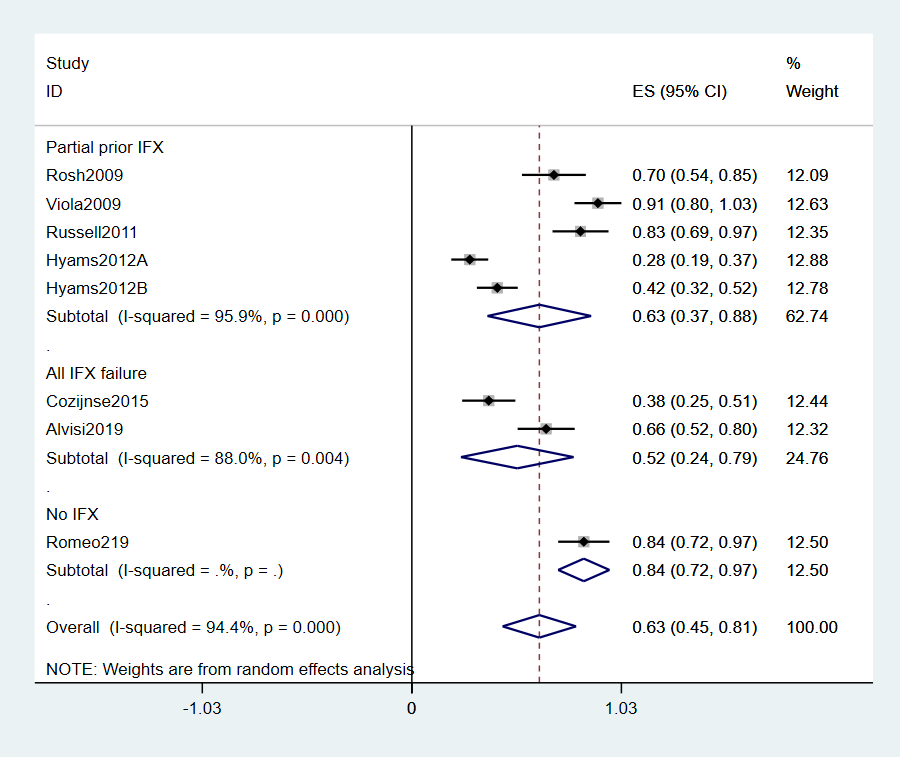

Supplement: Supplementary file 1 — Supplementary file1 (DOCX 1648 KB) [file 228_2023_3613_MOESM1_ESM.docx]
